# Wine in the Different Forms of Anœmia and Atonic Gout

**Published:** 1878-02

**Authors:** 


*Wine in the Different Forms of Anœmia and Atonic Gout.*—By M. E. Begin.

A translation from the French advocating the use of wine in these affections, especially French wines.

